# Toxic Metals Enrichment in the Surficial Sediments of a Eutrophic Tropical Estuary (Cochin Backwaters, Southwest Coast of India)

**DOI:** 10.1100/2012/972839

**Published:** 2012-05-03

**Authors:** G. D. Martin, Rejomon George, P. Shaiju, K. R. Muraleedharan, S. M. Nair, N. Chandramohanakumar

**Affiliations:** ^1^Department of Chemical Oceanography, School of Marine Sciences, Cochin University of Science & Technology, Cochin 682016, India; ^2^National Institute of Oceanography, Regional Center, P.O. Box 1913, Cochin 682018, India

## Abstract

Concentrations and distributions of trace metals (Cd, Co, Cr, Cu, Fe, Mn, Ni, Pb, and Zn) in surficial sediments of the Cochin backwaters were studied during both monsoon and pre-monsoon periods. Spatial variations were in accordance with textural charaterstics and organic matter content. A principal component analysis distinguished three zones with different metal accumulation capacity: (i) highest levels in north estuary, (ii) moderate levels in central zone, and (iii) lowest levels in southern part. Trace metal enrichments are mainly due to anthropogenic contribution of industrial, domestic, and agricultural effluents, whose effect is enhanced by settling of metals due to organic flocculation and inorganic precipitation associated with salinity changes. Enrichments factors using Fe as a normalizer showed that metal contamination was the product of anthropogenic activities. An assessment of degree of pollution-categorized sediments as moderately polluted with Cu and Pb, moderately-to-heavily polluted with Zn, and heavily-to-extremely polluted with Cd. Concentrations at many sites largely exceed NOAA ERL (e.g., Cu, Cr, and Pb) or ERM (e.g., Cd, Ni, and Zn). This means that adverse effects for benthic organisms are possible or even highly probable.

## 1. Introduction

Estuarine sediments constitute a fundamental step in the pathway of contaminants to the ocean as estuaries filter the fluvially fluxed metals derived from both natural and anthropogenic sources [1]. Since sediments often constitute the ultimate depository for trace metals introduced into aquatic systems, their analysis offers significant advantages over water analysis for the assessment and monitoring of metal contamination in estuaries, assuming that those metals are substantially not mobilized following the deposition [2–4]. Therefore, metal concentrations and distributions in sediments can provide the best information about spatial extent as well as magnitude of human-induced chemical change of the environment and may be useful indicators of contaminant related biological stress in estuarine ecosystems [5]. It follows that the distribution of total trace metals contents in estuarine sediments provides a simple means of expressing a measure of environmental pollution [6]. However, it is difficult to evaluate the relationships with the river inputs because the biogeochemical reactions in an estuary are complicated and not fully understood.

Pollution by toxic metals is one of the major threats to the estuarine ecosystem. However, despite the high concentration of industries and the consequent discharges of wastewater effluents into the Cochin estuary, very few studies have been carried out so far on assessing their impacts and the extent to which estuarine sediments have been contaminated by metal-rich waste discharges [7]. Though a few studies on trace metal distributions in water, particulates, and sediments are available, they are mainly concentrated on the northern part of the Cochin estuarine system. Owing to domestic and industrial pollutions, higher concentrations of Fe, Mn, Cu, and Zn were reported for the suspended particles in the Cochin backwaters [8]. Dissolved metal-salinity relationships in the Cochin Estuary revealed a large removal of metals from dissolved into particulate forms with increasing salinity from the monsoon to non-monsoon periods, due to processes of precipitation, adsorption, and flocculation [9]. The meandering flow in the perennially undulating water bodies or null zones of the Cochin backwaters induces faster coagulation or coprecipitation of dissolved metals as colloids in association with iron hydroxides by ion exchange processes under fluctuating salinity related to estuarine mixing [10]. The weak flushing in the null zones with relatively long water residence times has resulted in an entrapment of fine colloidal particles carrying trace metals loads that settled to the bottom thus increasing the sediment metal contents [9, 11]. Long-term trends in the metal contamination of sediments of the northwest Cochin backwaters showed a 3-fold enrichment for Fe, Cu, and Pb, 10-fold enrichment for Cd, and 25-fold enrichment for Zn, placing the estuary among the most impacted in the world [10]. These evidences underlined the need of a detailed study of the biogeochemical cycles of trace metals in the Cochin Estuary with emphasis on the driving processes. Therefore, the aim of the present study was to evaluate the spatial and seasonal variations of trace metals in sediments and their relationships especially with organic matter contents. In this context, the hydrological parameter (salinity), texture, organic carbon, and trace metals (Fe, Mn, Co, Cr, Ni, Cu, Zn, Cd, and Pb) concentrations in sediments were determined at 56 stations covering the entire Cochin Estuary during the monsoon and premonsoon periods. Additionally, pollution degrees were calculated using enrichment factor, contamination factor, and geoaccumulation index [6, 12].

## 2. Study Area

**Figure 1:**
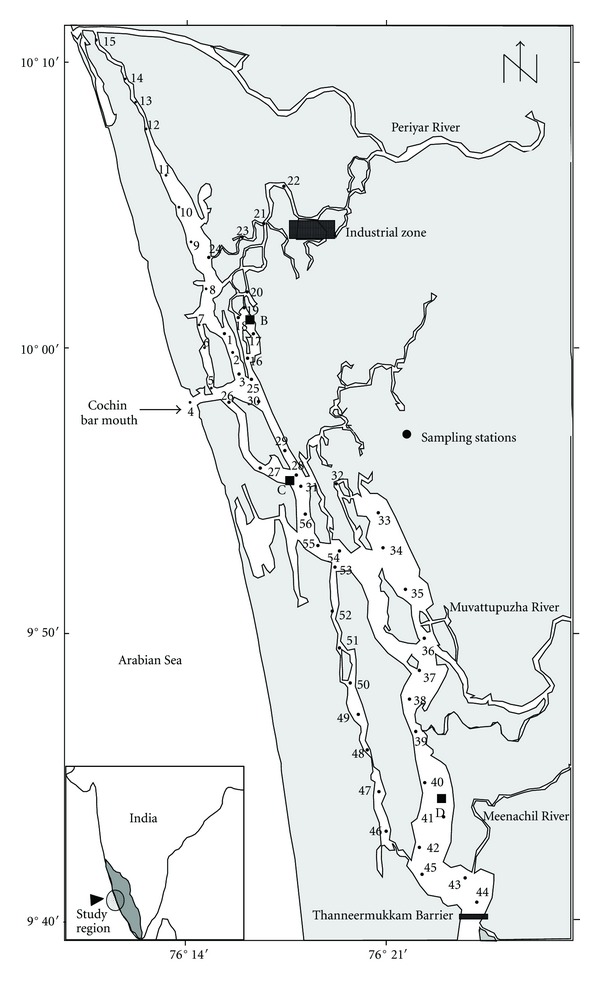
Map of the Cochin estuary with sampling locations.

The Cochin Estuary (Lat. 9°30′–10°10′ N and Lon. 76° 15′–76° 25′ E) extends between the cities of Azhikode in the north and Alleppey in the south, running parallel to the Arabian Sea (Figure 1). The estuarine system has two permanent openings, one at Cochin bar and the other at Azhikode. The Cochin bar mouth is the widest (450 m) and forms the main entrance to the Arabian Sea. The CE is generally wide (0.8–1.5 km) and deep (4–13 m) towards south but becomes narrow (0.05–0.5 km) and shallow (0.5–3.0 m) in its northern part. Six rivers (Pamba, Ackancovil, Manimala, Meenachil, Periyar, and Muvattupuzha) with their tributaries, along with several canals, bring large volumes of freshwater into the estuary. Among these rivers, Periyar (from north) and Muvattupuzha (south) discharge into the estuarine system and hence have an active influence on the prevailing salinity of the Cochin estuary. Tidal intrusion from the Arabian Sea (tidal range avg. 1 m) contributes a regular flow of salt water, which diminishes considerably towards the head of the estuary [14].

The microtidal (≤1.0 m) Cochin Estuary (CE) coastal system is the largest estuarine system (256 km^2^) in the south-west coast of India. It is fed by six rivers discharging about 2 × 10^10^ m^3^ y^−1^ of fresh water, >60% of which during the summer monsoon (June–September), 10–25% in the winter monsoon (November-December) [15, 16]. The human intervention in the Cochin Estuary dates back to 1836 but has accelerated during the last five decades. The booming city of Cochin, which is the largest in the west coast of India after Mumbai, has a population density of 6277 people per km^2^ and is recognized as one of the 17th major industrial cities of India by the World Bank. Export and related industrial activities are important contributors of the Cochin economy that can take advantage of the 4th largest port in India. This facility currently handles export and import of container cargos (1225 vessels, 13.9 × 10^6^ tons during 2005-06) at its terminal at the Willingdon Island. The city also hosts the principal chemical industries of the Kerala state (~70%) that are mainly located on the banks of the rivers Periyar and Chitrapuzha. There are ~250 industries, including Fertilizers and Chemicals Travancore Limited (FACT), Travancore Cochin Chemicals, Travancore Rayon's, Indian Rare Earths Limited, Hindustan Insecticides Limited ((HIL) the world's largest manufacturer of DDT), Cochin Refineries, Minerals and Rutiles Plant, Zinc-Smelter Plant, Hindustan Organic Chemicals Limited, Periyar Chemicals, United Catalysts, and Cominco Binani Zinc [16]. They manufacture a range of chemicals and petrochemical products, which include fertilisers, pesticides, rare-earth elements, rubber processing chemicals, zinc/chrome products, paints, pigments, phenolics, acids, batteries, oil, greases, mercury, and leather products. Many of these industries are 50 years old and employ highly polluting technologies. These industries take large amounts of fresh water from the river Periyar and in turn discharge 260 million liters of concentrated toxic effluents daily after little treatment [17]. Besides, the estuary receives untreated effluents (104 billion liters per day) from domestic sectors [18]. In addition, wastes from aquaculture fields (62 km^2^), agricultural fields (80 km^2^), coconut husk retting yards, fish processing plants, and animal bone processing units have increased the organic pollution in the estuary [19]. The continuous discharge of effluents from both domestic and economic sectors caused eutrophication of the estuarine waters, significantly increased organic carbon concentrations in sediments (four fold in last 4 decades), and affected the distribution of benthic fauna [20]. Even though the impact of historical and contemporary anthropogenic discharges has given rise to an enrichment on the essential metals like Fe, Mn, Co, Cr, Ni, Cu, and Zn and the nonessential metals like Cd and Pb in sediments of the Cochin Estuary, the elements of greatest concern are Cu, Zn, Cd, and Pb due to their elevated concentrations and potential toxicity to estuarine biota [10].

The annual rainfall at Cochin is around 3200 mm, 75% of which nearly occurring in summer monsoon periods, from June to September [20, 21]. Salinity remains at near-zero values over the surface layers of the CE during this period. In this “bar-built estuary”, seasonal effects of freshwater are readily visible in the prevailing salinities, which play an important role in the ecobiology of the estuarine system [7]. During presummer monsoon period (January–May), freshwater input to Cochin backwater is minimum due to low rainfall over the region. Hence, a gradient of salinity develops from the mouth to the head of the estuary and the lower reaches of the estuary behave as a section of the Arabian Sea [7, 22]. However, since the estuarine system is geographically located in the tropical region, seasonal mean temperature at the surface is about 28°C in summer monsoon periods (June–September) and 30°C in premonsoon periods (January–May) [7, 23].

## 3. Materials and Methods

Based on the above knowledge in seasonality, water and sediment samples were collected from 56 stations covering the entire estuary during February and September 2005 (Figure 1). The bottom water samples were collected using Niskin sampler, and salinity was measured using an electrodeless salinometer (Digi Auto3G, accuracy ±0.001). Textural characteristics were determined by pipette analysis [24]. Finely powdered and dried (~70°C) sediments were digested in a mixture of HF–HClO_4_–HNO_3_ [25] and brought into solution by 0.5 M HCl (25 mL) in Milli-Q water. Samples were analyzed on a flame AAS (PE AAnalyst 100) after calibration with suitable E-Merck elemental standards. Precisions of the analytical procedure were checked using a triplicate analysis of a certified reference material (BCSS-1) from the National Research Council of Canada. Precisions were typically 4% for Cu, Zn, Cd, and Pb, 9% for Cr, Co, and Mn, and 11% for Ni (Table 1). The results were mostly comprised within the uncertainty associated to certified concentration. For the estimation of organic carbon, the freeze-dried, powdered, sieved, and homogenized sediment was acidified (50% HCl) and gently warmed to remove carbonates. The organic carbon and nitrogen contents of the samples were estimated (in duplicate) in elemental analyser (Thermo Finnigan, Flash EA1112) using L-cystine as standard. The precision of the analysis checked against standard reference material (NIST 1941B) was found to be 0.4 ± 0.1% for C.

In the absence of sequential extraction of metals in sediments, principal component analysis (PCA) using PRIMER 5.1 will be used for making inferences on the source apportionment and important pathways of elemental deposition in the estuary [11].

## 4. Results and Discussion

### 4.1. Hydrography

**Figure 2:**
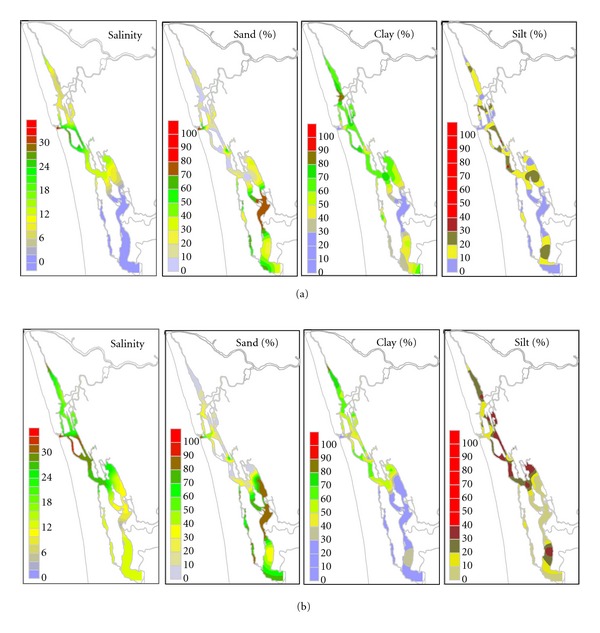
Salinity, sand, slit, clay during monsoon (a) and premonsoon (b).

The CE exhibited strong variations in flow and salinity characteristics with relatively high flow during the monsoon when compared to premonsoon conditions (Figure 2). Irrespective of seasons, high flow persisted in the central estuary, whereas, in the north and south estuary, a weak flow was observed [26, 27]. The salinity structure followed the same pattern, with variations over a wider interval during the monsoon (0–32.4) in comparison to premonsoon (2.61–33.3). The more saline waters were found near to bar mouth. The central estuary is highly saline with high- and low-level fluctuations noted in salinity (12.20–28.88 and 25.03–32.52, resp.) whereas the northern and southern estuaries (average values 8.42 and 1.34) are low and moderately (average values 18.88 and 11.97) saline, respectively, during the monsoon and premonsoon periods. During both seasons, weak flow in the north and south estuaries leads to low and moderate variations in salinity whereas high flow in the central estuary leads to high variations [20, 26].

### 4.2. Sediment Texture, Organic Carbon (SOC), and Trace Metals

**Figure 3:**
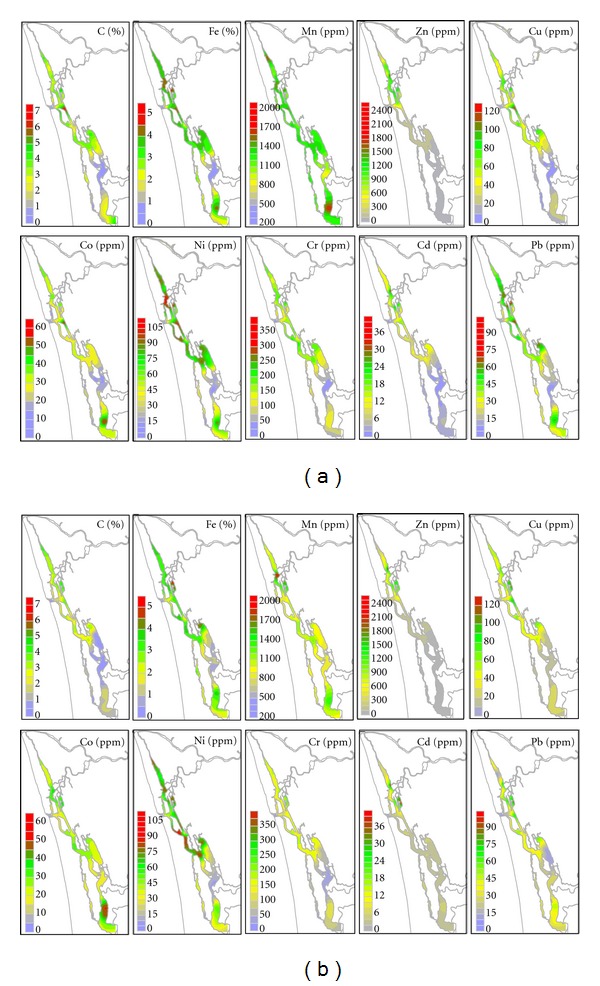
(a) Organic carbon and trace metal distribution during monsoon. (b) Organic carbon and trace metal distribution during premonsoon.

Sediment texture and organic carbon exhibited strong spatial and seasonal variability (Figures 2 and 3). The highest values of organic carbon were observed during the monsoon. The diversity from the Mandovi estuary [28] could be due to the differences in sediment grain size. During the monsoon sand, slit and clay content in sediments of the whole estuary varies in the ranges 0.26–79.5%, 0.1–37%, and 0–92% whereas during the premonsoon it varies in the ranges 0.16–91.03%, 0.17–41.36%, and 8.5–84%, respectively. During the monsoon sand, slit and clay content in sediments of the whole estuary averages to 33.26%, 12.14%, and 55.37% whereas during the premonsoon it averages to 39.57%, 17.74% and 42.53% respectively. During both seasons, sediment texture is dominated by clays. Similarly, during the monsoon sand, slit and clay content in sediments of the north estuary varies in the ranges 0.41–68.54%, 0.80–37%, and 32–92% whereas during the premonsoon it varies in the ranges 0.62–60.86%, 10.03–37.04%, and 28.5–73.5%, respectively. During the monsoon sand, slit and clay content in sediments of the north estuary, averages to 24.91%, 13.57%, and 63.0% whereas during the premonsoon it averages to 20.85%, 23.51%, and 55.19%, respectively. During the monsoon sand, slit and clay content in sediments of the central estuary varies in the ranges 0.26–74.75%, 0.57–32.35%, and 24.50–82.5% whereas during the premonsoon it varies in the ranges 0.16–77.34%, 0.45–41.36%, and 22.0–84.0%, respectively. During the monsoon sand, slit and clay content in sediments of the central estuary, averages to 20.65%, 15.66%, and 63.69% whereas during the premonsoon it averages to 19.06%, 25.47%, and 55.47%, respectively. Similarly, during the monsoon sand, slit and clay content in sediments of the south estuary varies in the ranges 15.7–79.5%, 0.10–30.41%, and 20.0–74.0% whereas during the premonsoon it varies in the ranges 30.06–89.72%, 0.17–38.44%, and 10.0–39.5%, respectively. During the monsoon sand, slit and clay content in sediments of the south estuary, averages to 53.04%, 7.25%, and 39.71% whereas during the premonsoon it averages to 73.3%, 6.37%, and 20.33%, respectively. During both seasons, a relatively high sandy environment prevails in the southern estuary and close to the bar mouth, whereas the northern and central estuary is dominated by clays. The relatively high concentrations of coarse sediment observed at bar mouth were due to estuarine bed-load movements associated with tides [29, 30]. The high silting environment throughout the estuary found during the premonsoon season is an indication of sedimentation processes associated with finer particles settled onto the bottom due to the prevailing of weak currents [16, 22].

Organic carbon content in estuarine sediments is controlled mostly by the rate of supply of terrestrial materials, rate of deposition of organic to inorganic constituents, primary productivity, and texture of sediments [31, 32]. Textural control over organic carbon is indicated by the correlations with sand, slit and clay percentages of sediments (Table 2, Figure 3). Organic carbon is found to be high during the monsoon (0.3–6.6%) whereas it is low during the premonsoon (0.8–4.3%). Seasonally, high variations were noted in the central estuary (avg. 3.5%—monsoon and avg. 2.76%—premonsoon) and southern estuary (avg. 1.71%—monsoon and avg. 1.00%—premonsoon), whereas the values remained steady in the northern estuary (avg. 3.27%—monsoon and avg. 3.24%—premonsoon). Organic carbon showed high positive correlations with both slit and clay and an inverse relationship with sand during both seasons. The positive relationship of organic carbon with slit and clay indicates its variable size-dependent scavenging in the north, central, and south estuaries. Various clay minerals adsorb substantial amount of organic matter formed by the decomposition of phytoplankton [31, 33]. An increase in organic carbon content during the monsoon when compared to premonsoon followed with a decrease in particle size (increase in slit and clay contents) of sediments is attributed to increase in surface area of fine particles. The high organic carbon associations coincided with high clay contents in the north and central estuary than the south estuary is attributed to enhanced adsorption of organic carbon onto clay minerals in the low- and high-salinity regimes than intermediate salinity.

 Trace metal variations in sediments of the whole estuary (Figure 3) during the monsoon and premonsoon are presented (Table 3). Iron and manganese showed different behaviors in the two periods (Figure 3). While Fe during the monsoon and premonsoon showed more or less similar concentrations in the three areas north (≥3.5%), central (≥3.2%), and south (≥2.0%) estuary, Mn decreased from the monsoon to premonsoon in the north (from ~1250 to ~1200 *μ*g g^−1^), central (from ~1280 to ~1000 *μ*g g^−1^), and south (from ~1200 to ~980 *μ*g g^*‒*1^) estuary. Co showed an equal higher accumulation (>25 ppm) behavior in the north, south, and central part of the estuary during both periods. Cr, Ni, Cu, and Pb showed similar patterns and trends, with higher accumulation in the north and central estuary (Cr ≥ 160 ppm, Ni ≥ 70 ppm, Cu ≥ 50 ppm, and Pb ≥ 44 ppm) when compared to the south estuary (Cr ≥ 60 ppm, Ni ≥ 25 ppm, Cu ≥ 12 ppm, and Pb ≥ 25 ppm) during the monsoon and premonsoon periods. Zn and Cd showed similar accumulation behaviors with the highest levels of accumulation towards the northern estuary (Zn ≥ 950 ppm and Cd ≥ 15 ppm) when compared to the central (Zn ≥ 140 and Cd ≥ 4 *μ*g g^−1^) and south (Zn ≥ 40 ppm and Cd ≥ 2 ppm) estuaries during the monsoon and premonsoon periods.

Metals such as Fe, Mn, Cr, Ni, Cu, Zn, Cd, and Pb generally exhibited higher levels in the northern and central estuary, with higher levels during the monsoon. The central estuary is reported to be dynamic, whereas the north and south estuaries showed flow restrictions and hence are more sensitive to the accumulation of contaminants [26]. Due to the weak flow and the huge input of industrial effluents, higher levels of trace metals were found in the northern estuary [19]. On the contrary, consistent with a strong flow, moderate levels of metals were found in the central estuary, which receives both domestic and industrial effluents during the monsoon period. Decreasing trends in metal levels, detected towards the central estuary during the premonsoon season, may be due to strong rectilinear current, which maintains an effective flushing [26]. Likewise, in relation with weak flow and minor inputs, lowest levels of trace metals characterised the southern estuary, which receives agricultural wastes from Kuttanad Paddy fields. When compared with other stations, bar mouth stations exhibited the lowest concentrations of all metals during the monsoon and premonsoon seasons. Here, seasonal variations were found to be marginal, which could be due to the constant mixing of fluvial sediments with marine sediments due to tidal action.

### 4.3. Trace Metal Contamination

**Figure 4:**
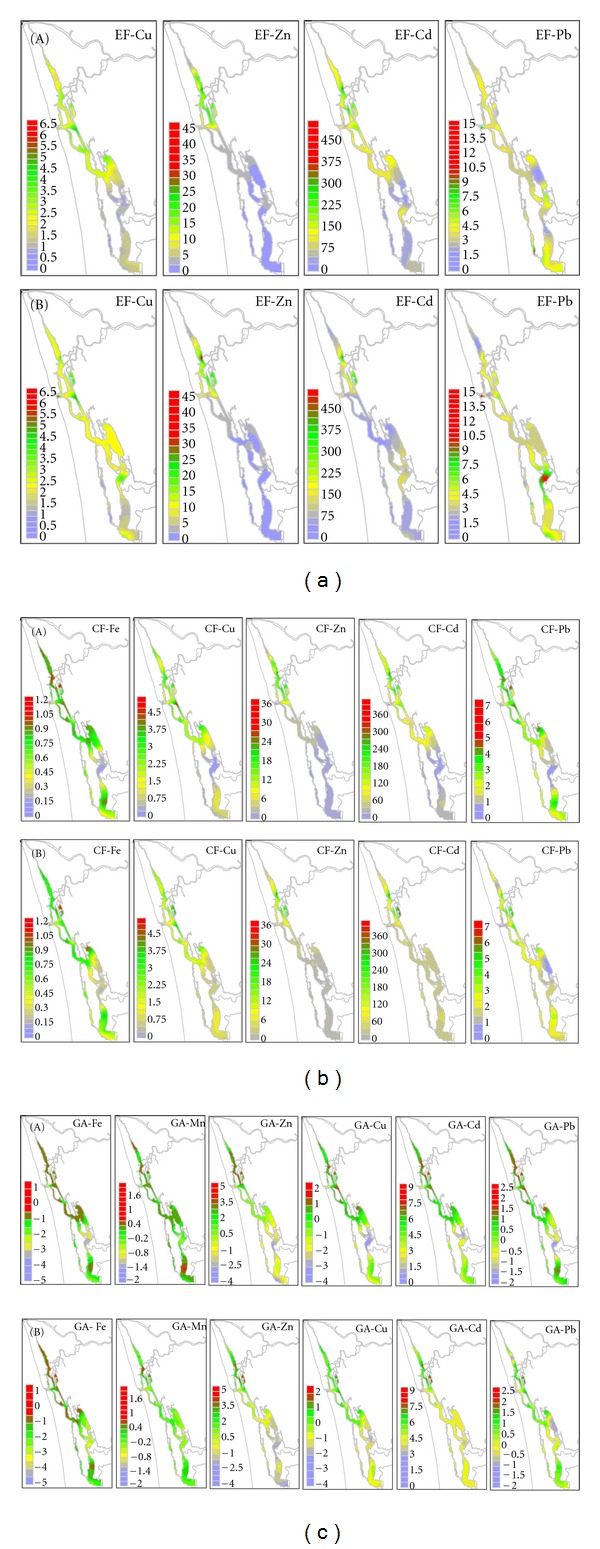
(a) Enrichment factor during monsoon (A) and premonsoon (B) season. (b) Contamination factor during monsoon (A) and premonsoon season (B). (c) Geoaccumulation index during monsoon (A) and premonsoon (B) season.

The degree of pollution in sediments can be assessed by determining the enrichment factor (EF), contamination factor (CF), and geo-accumulation index (*Igeo*). Variations of CF, EF, and *Igeo* along the estuary are shown (Figures 4(a)–4(c)).

#### 4.3.1. Enrichment Factor

In the present study enrichment factor was used to assess the level of contamination and the possible anthropogenic impact in sediments of the Cochin estuary. To identify anomalous metal concentration and to evaluate abundance of metals, geochemical normalization of the trace metals data to a conservative element, such as Al, Fe, and Si, was employed. In this study iron has also been used as a conservative tracer to differentiate natural from anthropogenic components. Iron has been chosen as normalization element because of its origin being exclusively lithospheric [34].

According to [35, 36], the metal enrichment factor (EF) is defined as follows:

(1) $$\text{Enrichment}\;\text{Factor}\;\left(\text{EF}\right)=\;\frac{C\;\left(\text{sample}\right)/\text{Fe}\left(\text{sample}\right)}{C\;\left(\text{crust}\right)/\text{Fe}\left(\text{crust}\right)},$$

where *C*_sample_ is trace element concentration in the sample, *C*_crust_ is trace element concentration in the continental crust [37], Fe_sample_ is Fe content in the sample, and Fe_crust_ is Fe content in the continental crust [37].

EF values were interpreted as suggested by [38] for metals studied with respect to natural background concentration. Many authors prefer to express the metal contamination with respect to average shale to quantify the extent and degree of metal pollution [39, 40]. In this study, the background concentrations of metals were taken from [37].  Figure 4(a) shows EF values of Cu, Zn, Cd, and Pb in the sediments of the Cochin Estuary. EF values were interpreted as the levels of trace metal pollution as suggested by [5] where EF < 1 indicates no enrichment, <3 is minor, 3–5 is moderate; 5–10 is moderately severe, 10–25 is severe, 25–50 is very severe, and >50 is extremely severe.

Fairly minor enrichment factor (2) of Cu as prevalent in the south estuary but gets increased to a moderate enrichment factor values of the ranges 4 to 5 in the north and central parts of the estuary during the monsoon period. Cu also showed moderate enrichment factor values of the ranges 3 to 5 in the south, central, and northern estuary during the premonsoon period. Zn showed moderate enrichment factors of 5 in the south and central estuary but is increased to severe enrichment factor values of the ranges 15 to 25 in the northern estuary during the monsoon and premonsoon seasons. Enrichment factors of Cd showed extremely severe enrichment factor values of the ranges 60 to 150 in the central and south estuaries, whereas extremely severe enrichment factor values of very higher ranges 200–350 were predominant in the northern estuary. Pb showed moderately severe enrichment factors in the ranges of 6 to 10 in the northern and southern parts of the estuary during the monsoon and premonsoon periods, respectively. Enrichment factors of Zn and Cd increase towards the upstream of the northern estuary, indicating increasing contamination from the industrialized zones of the river Periyar.

In order to evaluate anthropogenic influences on the sediments, [41] recommended EF values as an assessment criterion. EF values in the ranges 0.5 to 1.5 suggested that the trace metals sources might be entirely from crustal materials or natural weathering process, while EF values > 1.5 suggested that a significant portion of trace metal was delivered from noncrustal materials or nonnatural weathering processes [41]. The authors in [42] also divided the metal pollution in sediments into different categories based on EF values. If EF ≤ 2, it suggested deficiency to minimal metal enrichment, and if a value of EF was greater than 2, it suggested various degrees of metal enrichment.

From Figure 2, it could be seen that the enrichment factor values of Cu, Zn, Cd, and Pb were >2 in most regions, showing a high anthropogenic impact on the trace metal concentration levels in the estuary. Therefore, it could be deduced that Cu, Zn, Cd, and Pb pollution in the Cochin Estuary might entirely come from anthropogenic processes according to the scale proposed by [42]. As a result, these four trace metals pollutions should be currently a major concern.

In comparison, the EF values of Cu and Pb during the two seasons were almost >2 in most samples, showing a moderate-to-moderately-severe anthropogenic enrichment. The EF values of Zn lie in the ranges 5 to 25, showing a moderate-to-moderately-severe anthropogenic enrichment. Cd showed EF values in the ranges 60 to 150, showing extremely severe anthropogenic enrichment. Compared with the assessment criteria proposed by Birch [38], since the EF values of Cu, Pb, Zn, and Cd in the sediments were larger than 2, the anthropogenic inputs of Cu and Pb were moderately significant. This indicated that pollution of Cu, Pb, Zn and Cd really occurred in the Cochin Estuary.

From Figure 3, it is found that the sampling sites with higher EF values of Cu, Zn, Pb and Cd were not homogeneously distributed but concentrated in the certain portions of the northern, central, and southern parts of the Estuary. It might be implied that Cu, Zn, Pb, and Cd pollution in the north and central might be correlated with the industrial and domestic sewage effluents from Cochin city whereas for the south estuary is associated with agricultural waste discharges from the Kuttanad Paddy fields. The moderately severe anthropogenic enrichment factor of Zn, which is mainly concentrated in the north estuary, is attributed to industrial effluents from the river Periyar.

Given the moderate enrichment of Cu, Pb, Zn, and Cd found in the Cochin estuarine sediments, it would be worth to compare the metal concentration values with previously reported and other large urban-coastal settings or recognized polluted areas (Table 4). In comparison to the ranges and mean values of Cu, Pb, Zn, and Cd reported worldwide, the maximum values of Cu, Pb, Zn, and Cd found in this study were of the same order of magnitude or even higher than those reported for other polluted estuaries, placing the region as one among the impacted estuaries around the world. The ranges of Zn and Cd concentrations (10.0 ppm–2233.0 ppm and 0.2 ppm–40.7 ppm) found in this study agree well within the ranges of Zn and Cd values reported for Thames estuary (219.0 ppm–1050.0 ppm and 1.30 ppm–9.8 ppm) and New York Harbor (188.0 ppm–244.0 ppm and 1.0 ppm–2.0 ppm). Similarly, the ranges of Pb concentrations (6.8 ppm–99.6 ppm) found in this study agree well within the ranges of average Pb values reported for New South Wales Estuary (21.0 ppm), Solvay Estuary (25.0 ppm), Port Phillip Bay (43.5 ppm), Mumbai Harbor (48 ppm), Forth Estuary (89 ppm), and Izmir Harbor (97.0 ppm). The ranges of Cu concentrations (3.6 ppm–123.5 ppm) found in this study agree well within the ranges of average Cu values reported for New South Wales Estuary (6.0 ppm), Solvay Estuary (7.0 ppm), Port Phillip Bay (25.0 ppm), Ganges Estuary (53.0 ppm), Humber Estuary (70.0 ppm), Forth Estuary (86.0 ppm), and Bremen Harbor (87.0 ppm). The maximum Cu (123.5 ppm), Pb (99.6 ppm), Zn (2233.0 ppm), and Cd (40.7 ppm) concentrations obtained in this study are quite agreeable for relatively polluted regions, with the reported averages of Cu for Mumbai Harbor (105 ppm), Pb for Izmir Harbor (97.0 ppm), Zn for Rhine Estuary (2900 ppm), and Cd for Rhine Estuary (45 ppm). The maximum concentrations of the metals Cu (123.5 ppm), Pb (99.6 ppm), Zn (2233.0 ppm), and Cd (40.7 ppm) found in this study are much higher than the maximum concentrations of metals Cu (53.2 ppm), Pb (71.3 ppm), Zn (1266.0 ppm), and Cd (14.9 ppm) previously reported by [17] suggesting that the magnitude of trace metal pollution in sediments of the Cochin backwaters has been increasing over the last few decades.

#### 4.3.2. Contamination Factor

The level of contamination of sediment by a metal is often expressed in terms of a contamination factor:

(2) $$\text{Contamination}\;\text{Factor}\;\left(\text{CF}\right)=\frac{\text{Metal}\;\text{content}\;\text{in}\;\text{sediment}}{\text{Back}\;\text{ground}\;\text{value}\;\text{of}\;\text{metal}},$$

where CF < 1 refers to low contamination, 1 ≤ CF ≤ 3 means moderate contamination, 3 ≤ CF ≤ 6 indicates considerable contamination, and CF > 6 indicates very high contamination [43]. A CF, calculated as the ratio between the sediment metal content at a given station and the normal concentration levels, reflects the metal enrichment in the sediment when CF > 1 for a particular metal, it means that the sediment is contaminated by the element, and if CF < 1, then there is no metal enrichment by natural or anthropogenic inputs. While calculating the CF of the sediments in the study area, we have taken the world crustal average contamination of the trace metals under consideration reported by of background values.

**Figure 5:**
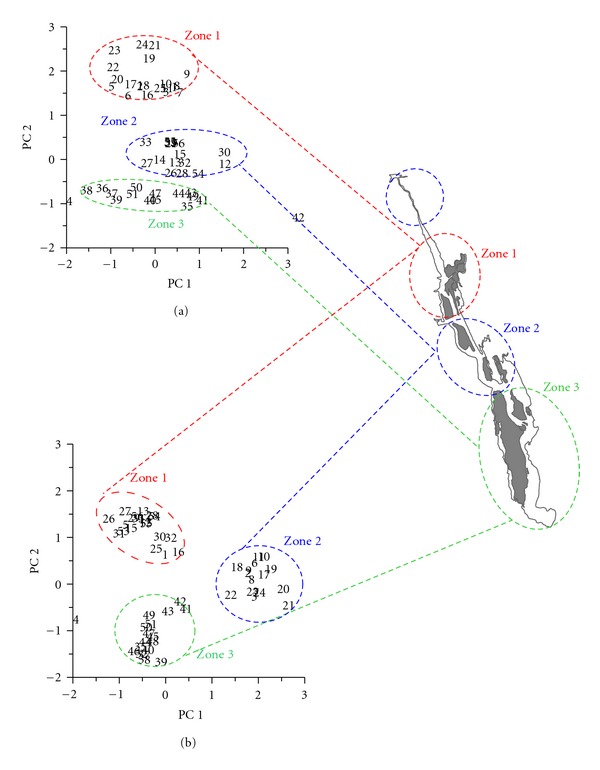
Principal component analysis during monsoon (a) and premonsoon (b).

The contamination factors for the trace metals Cu, Zn, Cd, and Pb in the bottom sediment of the Cochin Estuary are presented in Figure 5, indicating a moderate-to-high level considerable contamination of the sediments by these trace metals. The CF value for Cu lies in the ranges 1 to 3 indicating a moderate level of contamination by this metal. CF values for Cu ≥ 2 was evident in the south and central estuary with a progressive increase of CF values ≥ 3 for the north estuary. The CF value for Zn lies in the ranges 4 to 16 indicating a considerable to very high level of contamination by this metal. CF values for Zn ~ 4 was evident in the south and central estuary with a progressive increase of CF values > 10 for the north estuary. Cd showed high CF values in the ranges 50 to 75 in the south and central estuary but gets increased to very high CF values in the ranges 100 to 200 in the north estuary indicating a very high level of contamination by this metal in the entire estuary. The CF value for Pb lies in the ranges 2 to 3 indicating a moderate level of contamination by this metal. Moderate CF values of ~3 were evident for Pb in the south, central, and north estuary.

#### 4.3.3. Geoaccumulation Index

To understand current environmental status and trace metal pollution extent with respect to natural environment, geoaccumulation index is used. The geoaccumulation index (*I*_geo_), introduced by [39], was used to assess metal pollution in sediments according to the equation *I*_geo_ = log_2_ (*C*_*n*_/1.5*B*_*n*_), where *C*_*n*_ is measured concentration of metal in the sediment, *B*_*n*_ is geochemical background value in average shale [44] of element *n*, and 1.5 is the background matrix correction in factor due to lithogenic effects.

The study [39] distinguished seven classes of geoaccumulation index in sediments, where *I*_geo_ < 0 refers to unpolluted, *I*_geo_  = 0-1 refers to unpolluted to moderately polluted, *I*_geo_  = 1-2 refers to moderately polluted, *I*_geo_  = 2-3 refers to moderately to heavily polluted, *I*_geo_ = 3-4 refers to heavily polluted, *I*_geo_  = 4-5 refers to heavily to extremely polluted, and *I*_geo_  > 5 refers to extremely polluted. In general, the sites are uncontaminated to extremely contaminated with respect to trace metals. The results of *I*_geo_ values are shown in Figure 3. From this classification criteria, all the sediments could be approximately categorized as practically unpolluted with Fe, Mn, Ni, Co, and Cr (*I*_geo_ < 0 for each trace metal), and moderately polluted with Cu and Pb (*I*_geo_ value 0 to 2 for both trace metals), moderately to heavily polluted with Zn (*I*_geo_ value 0 to 3), and heavily to extremely polluted with Cd (*I*_geo_ value 4 to 6), respectively.

### 4.4. Factors Controlling the Distribution of Trace Metals in Cochin Estuary

#### 4.4.1. Correlation Analysis

The strong positive interrelationships among all the metals in sediments (Table 2) indicate that the northern, central, and southern parts of Cochin Estuary are influenced by point source contaminants [19]. Moreover, the total trace metal concentration in sediments are influenced by organic carbon and texture. Organic carbon, slit, and clay contents in sediments showed positive correlations with all trace metals. Both slit and clay showed high positive correlations with all trace metals. Nevertheless, moderate elemental associations are noted for all elements with slit content, but better level of correlations was depicted with clay content. A highly significant correlation of metals with organic carbon content discloses its association with organic molecules in the north, central, and south estuary. This is in agreement with the reported high chlorophyll concentrations in the water column and high organic matter content in the sediments [20]. Previous works however did not find any strong associations of metals with organic carbon in sediments of the Cochin estuary, and hence it is still a matter of controversy that whether metal distributions result from grain size or organic matter content [18, 30]. However, significant metal associations with organic matter were noted in Indian estuaries as well as worldwide [6, 31, 58, 59]. The spatial representation of correlations of metals in sediments of Cochin Estuary is controlled primarily by the association of metals with fine-grained, organic-rich phase, accumulated in the north, central, and south estuary with high, moderate, and low intensities. These accumulations are related to anthropogenic inputs from industrial, domestic, and agricultural effluents, which are further triggered by the complexing nature of organic matter [60]. Thus, metal associations with organic carbon in sediments suggest a strong interaction of metal ions with organic matter, which in turn are further concentrated by adsorption onto clays [61].

Another factor that leads to trace metal enrichment in sediments is scavenging reactions involving hydrous and hydroxy-oxides of Fe and Mn, which constitute significant sinks of trace metals in aquatic environments [62, 63]. In estuarine water bodies, the effective binders for elements, such as Fe-Mn oxides, either in bulk phases or as coatings of mineral particles readily adsorbs whereas organic matter flocculates, which are consequently sunk to sediments [58, 64–69]. The strong Fe-Mn intermetallic relationship in sediments reveals formation of Fe-Mn oxide geochemical phases and significant correlations of all metals with organic carbon (Table 2) indicate organic associations during its transport in the estuarine environment [6, 70]. Fe and Mn exhibited strong correlations with the metals Zn, Co, Cr, Ni, Cu, Pb, and Cd in zone 1 and zone 3 during the monsoon. These strong correlations are also evident in zone 2 for all the metals (except for Pb and Cr with Mn a weak correlation is noted) during the monsoon. However, Mn showed weak correlations for the metals Cu, Zn, Cd, and Pb in zone 1, Cr, Zn, Cd, and Pb in zone 2 and Zn and Cd in zone 3, respectively. The strong correlations of Fe and organic carbon with all other metals are relevant in the three zones, which indicates that Fe plays a major role in controlling the adsorption and flocculation of all other elements during the monsoon and premonsoon seasons. Despite significant correlations, the behavior of Fe, Mn, and organic carbon towards metals (Cu, Zn, Cd, and Pb) was found to be quite contrasting in Z_2_ during the monsoon and premonsoon seasons. Thus, a significant fraction of trace metals is flocculated along with organic matter or gets adsorbed onto Fe-Mn oxide geochemical phases controlling the trace metals in the sediments.

#### 4.4.2. Principal Component Analysis (PCA)

PCA was performed on the entire data set of salinity, texture, organic carbon, and trace metals in order to identify the major estuarine process that leads to the sediment trace metal enrichment during the monsoon and premonsoon seasons (Figure 5). Three homogenous groups of clusters based on salinity and geochemical compositions were identified for monsoon and premonsoon seasons. A first cluster characterized with low salinity, high levels of slit, clay, organic carbon, and trace metals represents 20 stations of the northern estuary (Z_1_). A second cluster showed high salinity and comparatively moderate levels of slit, clay, organic carbon and trace metals, represents 16 stations of the central estuary (Z_2_). Similarly, a third cluster characterized with moderate salinity and low levels of slit, clay, organic carbon and trace metals represents 18 stations in the south estuary (Z_3_). Both monsoon and premonsoon seasons yielded more or less identical clusters, as was evident from the figure that 80% of stations were identical for zones (Z_1_ and Z_2_) and other stations remained same as in zone (Z_3_) (Figure 5).

### 4.5. Possible Biological Effects

Since India has no established sediment quality guidelines at this time, the US National Oceanic and Atmospheric Administration (NOAA) guidelines were used as interim measures to assess whether the concentrations of trace metals in sediments could have adverse biological impacts. The authors in [13] suggested two guideline values, namely, the ER-L (effective range: limit) and ER-M (effective range: medium) delineating three concentration ranges of a particular metal (Table 5). If a metal occurs in concentrations below ER-L value, effects on the biota would rarely be observed. At concentrations ≥ER-L but <ER-M, the biota could “occasionally” be affected by the pollutant, whereas at concentrations ≥ER-M, effects would be expected to occur “frequently.” Accordingly, in the estuarine sediments that are mostly confined to the north estuary, 25 stations for Zn, 29 stations for Cd, and 34 stations for Ni exceeded the NOAA guideline ER-M values. Similarly, as seen in Table 5, 33 stations for Cu, 41 stations for Pb, and 40 stations for Cr exceeded the NOAA guideline ER-L values. This indicates that the existing concentrations of metals (Zn, Cd, and Ni) in the sediments are sufficiently high to cause adverse biological effects.

## 5. Conclusions

The elevated *I*_geo_ values identified for Cu, Pb, Zn, and Cd in the Cochin Estuary indicate that surface sediments are moderately polluted with Pb and Cu, moderately to heavily polluted with Zn, and heavily to extremely polluted with Cd to some extent, probably because of anthropogenic activities. The magnitude of trace metal pollution in sediments of the Cochin backwaters has been increasing over the last few decades and was regarded as a product of anthropogenic contamination. However, the present trace metal concentrations have not yet reached levels that could be considered “extreme.” However, poor sediment flushing conditions, enclosed nature of the area, and suitable adsorption of metals in the sedimentary compartments (slit, clay, and organic carbon) suggests it as a sensitive ecosystem. Unfortunately, even though environmental protection measures are improving, the rate of growth of the Cochin city (*2nd-tier metro city in India*) suggests that industrial, domestic, and agricultural pollutant sources are likely to cause increasing problems in the future. Of particular concern are organic pollution, eutrophication, and reclamation, which pose threats for the future of Cochin backwaters, which was once regarded as a “pristine” resource. This study provides a baseline data for future research on anthropogenic impacts in the region.

## Figures and Tables

**Table 1:** Trace metal extracted from the standard reference material BCSS-1.

	Trace metal extracted from the standard reference material BCSS-1 (*n* = 5)								
Concentration (ppm)	Cu	Co	Cr	Pb	Ni	Fe	Zn	Mn	Cd
Metal extracted^a^	18.5	11.4	123	22.7	55.3		119	229	0.300
Metal extracted^b^	18.2	10.3	133	21.8	61.1	29200	114.6	209.8	0.291
Accuracy (± %)	2	9	9	4	11		4	8	3

^a^Certified values corresponding to the total extraction of trace metals from the standard reference material BCSS-1.

^b^Values of the metals extracted from the standard reference material BCSS-1 in the present study.

**Table:** (a)

						Monsoon								
	Salinity	Sand	Slit	Clay	C	Fe	Mn	Zn	Cd	Pb	Cu	Ni	Cr	Co
Salinity	1.00													
Sand	0.23	1.00												
Slit	−0.21	−0.60	1.00											
Clay	−0.32	−0.88	0.35	1.00										
C	−0.53	−0.69	0.65	0.70	1.00									
Fe	−0.62	−0.75	0.52	0.79	0.82	1.00								
Mn	−0.08	−0.60	0.46	0.56	0.68	0.68	1.00							
Zn	−0.53	−0.39	0.43	0.49	0.76	0.74	0.66	1.00						
Cd	−0.52	−0.45	0.44	0.49	0.74	0.70	0.60	0.93	1.00					
Pb	−0.37	−0.52	0.45	0.54	0.78	0.76	0.69	0.73	0.77	1.00				
Cu	−0.42	−0.53	0.43	0.63	0.85	0.71	0.77	0.81	0.80	0.82	1.00			
Ni	−0.41	−0.81	0.63	0.80	0.76	0.90	0.75	0.65	0.60	0.64	0.68	1.00		
Cr	−0.51	−0.64	0.47	0.71	0.85	0.75	0.65	0.71	0.73	0.79	0.95	0.72	1.00	
Co	−0.38	−0.50	0.38	0.58	0.62	0.73	0.65	0.59	0.57	0.72	0.61	0.66	0.54	1.00

						Premonsoon								
	Salinity	Sand %	Clay %	Silt %	C	Fe	Mn	Zn	Cd	Pb	Cu	Ni	Cr	Co

Salinity	1.00													
Sand %	−0.04	1.00												
Clay %	0.03	−0.82	1.00											
Silt %	−0.02	−0.76	0.38	1.00										
C	−0.61	−0.53	0.42	0.50	1.00									
Fe	−0.38	−0.63	0.56	0.55	0.87	1.00								
Mn	−0.21	−0.39	0.56	0.10	0.63	0.67	1.00							
Zn	−0.53	−0.53	0.36	0.56	0.82	0.69	0.42	1.00						
Cd	−0.56	−0.43	0.22	0.57	0.86	0.72	0.44	0.87	1.00					
Pb	−0.69	−0.18	0.00	0.33	0.82	0.60	0.41	0.81	0.81	1.00				
Cu	−0.58	−0.32	0.21	0.45	0.78	0.62	0.46	0.75	0.72	0.86	1.00			
Ni	0.04	−0.78	0.75	0.62	0.52	0.66	0.67	0.45	0.47	0.23	0.35	1.00		
Cr	−0.38	−0.34	0.33	0.29	0.68	0.65	0.50	0.70	0.52	0.67	0.76	0.26	1.00	
Co	−0.42	−0.44	0.48	0.27	0.82	0.76	0.79	0.61	0.60	0.67	0.65	0.54	0.66	1.00

**Table:** (b)

						Monsoon								
	Salinity	Sand	Slit	Clay	C	Fe	Mn	Zn	Cd	Pb	Cu	Ni	Cr	Co
Salinity	1.00													
Sand	0.21	1.00												
Slit	−0.05	−0.81	1.00											
Clay	−0.28	−0.90	0.48	1.00										
C	−0.06	−0.69	0.51	0.65	1.00									
Fe	−0.20	−0.88	0.62	0.86	0.81	1.00								
Mn	−0.22	−0.68	0.57	0.60	0.73	0.72	1.00							
Zn	0.00	−0.52	0.43	0.46	0.86	0.67	0.73	1.00						
Cd	0.05	−0.49	0.39	0.45	0.90	0.70	0.70	0.87	1.00					
Pb	−0.01	−0.57	0.35	0.60	0.78	0.76	0.42	0.72	0.74	1.00				
Cu	0.01	−0.57	0.47	0.51	0.87	0.73	0.60	0.89	0.86	0.89	1.00			
Ni	−0.26	−0.88	0.58	0.89	0.78	0.93	0.61	0.65	0.61	0.73	0.68	1.00		
Cr	−0.31	−0.78	0.45	0.83	0.46	0.84	0.43	0.31	0.27	0.52	0.36	0.87	1.00	
Co	−0.09	−0.42	0.40	0.33	0.76	0.58	0.62	0.90	0.68	0.59	0.77	0.56	0.26	1.00

						Premonsoon								
	Salinity	Sand	Slit	Clay	C	Fe	Mn	Zn	Cd	Pb	Cu	Ni	Cr	Co

Salinity	1													
Sand	0.26	1												
Slit	−0.39	−0.84	1											
Clay	−0.11	−0.92	0.55	1										
C	−0.24	−0.89	0.71	0.84	1									
Fe	−0.47	−0.89	0.83	0.75	0.79	1								
Mn	−0.21	−0.65	0.55	0.60	0.57	0.66	1							
Zn	−0.18	−0.60	0.69	0.42	0.79	0.61	0.41	1						
Cd	−0.23	−0.59	0.48	0.56	0.63	0.72	0.36	0.58	1					
Pb	−0.04	−0.44	0.58	0.25	0.52	0.64	0.44	0.73	0.47	1				
Cu	−0.24	−0.64	0.77	0.42	0.76	0.76	0.51	0.90	0.63	0.84	1			
Ni	−0.26	−0.94	0.79	0.86	0.84	0.83	0.70	0.53	0.50	0.44	0.59	1		
Cr	−0.30	−0.96	0.77	0.90	0.85	0.88	0.73	0.54	0.56	0.47	0.60	0.98	1	
Co	−0.48	−0.45	0.40	0.39	0.35	0.68	0.71	0.18	0.37	0.43	0.37	0.56	0.59	1

**Table:** (c)

						Monsoon								
	Salinity	Sand	Slit	Clay	C	Fe	Mn	Zn	Cd	Pb	Cu	Ni	Cr	Co
Salinity	1.00													
Sand	−0.26	1.00												
Slit	0.26	−0.78	1.00											
Clay	0.21	−0.92	0.47	1.00										
C	0.12	−0.87	0.49	0.92	1.00									
Fe	0.32	−0.79	0.80	0.60	0.65	1.00								
Mn	0.23	−0.71	0.78	0.51	0.57	0.90	1.00							
Zn	0.41	−0.79	0.66	0.69	0.67	0.84	0.70	1.00						
Cd	0.29	−0.81	0.79	0.64	0.78	0.73	0.69	0.56	1.00					
Pb	−0.11	−0.72	0.66	0.59	0.63	0.70	0.80	0.58	0.54	1.00				
Cu	0.31	−0.86	0.87	0.67	0.74	0.85	0.78	0.79	0.85	0.64	1.00			
Ni	0.20	−0.67	0.76	0.46	0.64	0.75	0.77	0.67	0.84	0.60	0.88	1.00		
Cr	0.46	−0.80	0.86	0.58	0.60	0.83	0.81	0.83	0.72	0.66	0.90	0.77	1.00	
Co	−0.11	−0.54	0.73	0.29	0.47	0.66	0.79	0.36	0.77	0.72	0.68	0.78	0.58	1.00

						Premonsoon								
	Salinity	Sand	Slit	Clay	C	Fe	Mn	Zn	Cd	Pb	Cu	Ni	Cr	Co

Salinity	1.00													
Sand	−0.25	1.00												
Slit	0.21	−0.96	1.00											
Clay	0.26	−0.91	0.75	1.00										
C	0.35	−0.73	0.72	0.63	1.00									
Fe	0.42	−0.79	0.83	0.61	0.77	1.00								
Mn	0.35	−0.82	0.73	0.82	0.62	0.66	1.00							
Zn	0.22	−0.59	0.63	0.45	0.65	0.51	0.38	1.00						
Cd	0.07	−0.60	0.66	0.42	0.70	0.66	0.40	0.52	1.00					
Pb	0.18	−0.66	0.71	0.48	0.70	0.62	0.60	0.47	0.31	1.00				
Cu	0.17	−0.88	0.84	0.81	0.77	0.66	0.78	0.57	0.58	0.65	1.00			
Ni	0.39	−0.87	0.82	0.81	0.85	0.79	0.80	0.70	0.69	0.53	0.88	1.00		
Cr	0.37	−0.89	0.85	0.81	0.83	0.70	0.79	0.66	0.62	0.65	0.89	0.88	1.00	
Co	0.14	−0.90	0.86	0.83	0.58	0.57	0.84	0.46	0.48	0.63	0.85	0.74	0.84	1.00

**Table 3:** Trace metals levels in different regions of the Cochin estuary (CE).

Metal	Entire CE min-max, (mean)		Northern CE min-max, (mean)		Central CE min-max, (mean)		Southern CE min-max, (mean)	
	Monsoon	Premonsoon	Monsoon	Premonsoon	Monsoon	Premonsoon	Monsoon	Premonsoon
Fe (%)	0.45 4.83, (3.02)	0.22–4.86, (2.80)	1.59–4.83, (3.52)	1.86–4.86, (3.53)	1.93–4.10, (3.54)	1.44–4.69, (3.19)	0.64–4.3, (2.12)	0.69–3.88, (1.88)
Mn (ppm)	370.0–1739.7, (1232.0)	380.0–1946.0, (1063.6)	983.0–1564.0, (1254.0)	775.0–1946.0, (1216.0)	1034.4–1605.4, (1280.0)	844.5–1256.9, (1011.8)	763.0–1739.7, (1209.0)	739.9–1435.8, (986.6)
Co (ppm)	12.0–58.4, (27.4)	3.4–51.2, (24.8)	13.2–42.3, (28.0)	8.89–40.28, (28.5)	16.2–32.2, (25.0)	13.2–42.2, (28.0)	4.3–51.2, (24.0)	4.25–51.2, (22.0)
Cr (ppm)	20.7–310.0, (131.0)	7.0–379.6, (132.8)	58.0–379.6, (188.9)	58.3–379.6, (188.9)	85.8–213.6, (168.9)	72.0–188.8, (151.0)	36.0–124.0, (65.3)	36.0–124.0, (65.3)
Ni (ppm)	8.5–103.7, (62.9)	2.9–91.8, (51.0)	33.9–103.7, (74.4)	42.3–81.5, (62.3)	37.0–98.3, (79.5)	31.3–91.8, (68.2)	11.0–90.5, (38.0)	3.5–54.4, (26.4)
Cu (ppm)	3.6–123.0, (43.0)	7.2–123.5, (43.7)	19.3–123.5, (56.7)	32.2–123.5, (66.6)	31.6–118.5, (61.6)	16.6–95.5, (49.2)	3.6–30.5, (14.3)	9.07–29.29, (17.0)
Zn (ppm)	10.0–1907.6, (422.5)	14.9–2233.0, (422.8)	132.9–1907.6, (984.9)	120.7–2233.2, (1024.9)	81.8–391.4, (184.6)	14.54–313.3, (146.3)	10.0–81.8, (49.0)	19.9–76.4, (41.9)
Cd (ppm)	0.2–34.0, (9.5)	0.94–40.7, (8.7)	0.8–34.0, (15.3)	3.1–40.7, (18.2)	5.5–18.5, (10.5)	2.5–6.1, (4.2)	0.2–4.8, (2.1)	1.8–6.2, (3.1)
Pb (ppm)	6.8–73.6, (39.0)	9.7–99.6, (40.5)	20.7–73.6, (48.7)	10.2–99.6, (53.8)	23.8–71.6, (45.4)	14.9–71.4, (41.6)	6.8–58.5, (25.9)	11.7–50.1, (27.9)

**Table 4:** Comparison of heavy metal levels (ppm) in the Cochin backwaters to that of other Indian and globally impacted coastal systems.

Location	Zn	Cd	Pb	Cu	References
Continental crustal average	52	0.1	14.8	104	[44]
Cochin estuary	10.0–2233.0	0.2–40.7	6.8–99.6	3.6–123.5	Present study
Cochin estuary	592–1266.0	6.2–14.9	39.9–71.3	32.4–53.2	[10]
Mumbai harbour, India	155	—	48	105	[45]
Ganges estuary, India	611	—	115	53	[46]
Bilbao estuary, N. Spain	1092	—	314	263	[47]
Humber estuary, UK	319	—	127	70	[48]
Solway estuary, Scotland	59	—	25	7	[49]
Forth estuary, Scotland	150	—	89	86	[50]
Thames estuary, UK	219–1050	1.3–9.8	179–1634	61–348	[51]
Port Phillip Bay, Victoria	—	2.37	43.5	25.0	[52]
New South Wales estuary, Australia	—	1.60	21.0	6.0	[53]
New York Harbor (USA)	188–244	1-2	109–136	105–131	[54]
Bremen Harbor (Germany)	790	6.0	122	87	[55]
Izmir Harbor (Turkey)	182	6.2	97	182	[56]
Rhine estuary (Germany)	2900	45	800	600	[57]

**Table 5:** Number of samples that had metal concentrations above and below the sediment effects data of ERM and ERL in the Cochin Estuary. ERM and ERL guidelines were from [13].

Metal	Zn	Cd	Cu	Pb	Cr	Ni
ER-L (mg kg^−1^)	15O	1.2	34	46.7	81	20.9
ER-M (mg kg^−1^)	410	9.6	270	218	370	51.6
Between ERL and ERM (no. of stations in CE)	11	21	33	41	40	16
Above ERM (no. of stations in CE)	25	29	0	0	3	34
Below ERL (no. of stations in CE)	20	6	23	15	13	6
